# Intentional Replantation of Single-Rooted and Multi-Rooted Teeth: A Systematic Review

**DOI:** 10.3390/healthcare11010011

**Published:** 2022-12-21

**Authors:** Massimo Pisano, Federica Di Spirito, Stefano Martina, Giuseppe Sangiovanni, Francesco D’Ambrosio, Alfredo Iandolo

**Affiliations:** Department of Medicine, Surgery and Dentistry “Scuola Medica Salernitana”, University of Salerno, Via S. Allende, 84081 Baronissi, Italy

**Keywords:** intentional replantation, intentional replantation procedure, intentional replantation case-report, single-rooted replantation, multi-rooted replantation

## Abstract

The technique of intentional replantation can provide a second chance to save teeth that would be destined for extraction. Therefore, the present systematic review aimed primarily to estimate tooth survival after intentional replantation and secondarily to compare treatment outcomes in single-rooted and multi-rooted teeth. The study protocol was developed before the analysis according to the Preferred Reporting Items for Systematic Review and Meta-Analyses guidelines. Articles were electronically searched in PubMed/MEDLINE, the COCHRANE library and Google Scholar by two independent reviewers, and those that met the eligibility criteria were included. A statistical analysis using the chi-square test with a *p*-value of <0.05 was performed on the reported outcomes of intentional replantation. A total of 44 single-rooted replanted teeth with five failures (11.36%) and 42 multi-rooted replanted teeth with six failures (14.28%) were reported in the literature, corresponding to a survival rate of 88.64% and 85.57%, respectively. The overall survival rate for the replantation procedure was 86.7%, indicating that intentional replantation can be considered a safe therapeutic choice, with no statistically significant difference between the survival rates of single-rooted and multi-rooted replanted teeth.

## 1. Introduction

Intentional replantation (IR) is a multistage surgical procedure based on the controlled extraction of a tooth and its subsequent repositioning in the original socket to perform root surface revision and subsequent endodontic treatment in the extra-oral environment [1].

Since surgical phase IR is considered the most technically delicate phase of the procedure [2], it must be performed with extreme precision and care to improve treatment outcomes and survival rates. Tooth extraction must be as atraumatic as possible to avoid both fractures of the tooth and, most importantly, damage to the periodontal ligament (PDL), which may play a critical role in healing and, consequently, treatment success [3].

Subsequently, the extracted tooth is carefully examined to assess possible fractures or anatomical features that require special attention, such as the presence of additional or accessory canals or multiple foramina [4], and accordingly decide whether to proceed with extra-oral endodontic treatment [5]. At this stage, PDL preservation as well as proper management of the tooth under extra-alveolar environmental conditions, which affect the overall treatment success, are crucial [6,7]. Since it has been found that extra-alveolar remaining in a dry environment longer than 15 min may affect the PDL conditions and consequently increase the risk of dental ankylosis after IR [8], it is recommended to keep the extra-alveolar time as short as possible and to preserve the tooth in a moist environment to improve the predictability of the procedure [9,10].

After extra-oral endodontic treatment, the original tooth socket must be prepared. Complete resection of cystic or granulomatous tissue from the dental alveolus (alveolar curettage) to promote healing of the alveolus is still controversial, as it would be particularly difficult to avoid simultaneous removal or at least damage to the PDL fibers that remained attached to the alveolar walls [11]; consequently, a healing technique for the apical part of the dental alveolus has been proposed, in which the entire inflammatory lesion is removed without affecting the walls of the affected pockets [12,13].

After the preparation of the alveolus, the tooth is carefully inserted with digital pressure in the axial direction of the alveolus. Some authors have suggested applying the patient’s bite to the tooth in case of resistance to replantation [14,15].

In the surgical phase, which involves the same procedure in single-rooted and multi-rooted teeth, the main difference between the two types of teeth is the atraumatic phase of extraction. The presence of intraradicular septa or anatomies with severe curvatures is an anatomical limitation for multi-rooted teeth, which pose greater surgical difficulties in the atraumatic extraction phase [5]. The preservation of the shape of the alveolus is also more complex in multi-rooted teeth. The final splinting of the replanted teeth is still controversial. A variety of splinting methods and materials have been reported, ranging from orthodontic wires to composite resins and sutures [11], with removal periods varying accordingly, from seven to ten days to three to four weeks.

The IR procedure is indicated when neither orthograde treatment nor apical surgery can be performed [1] and when a symptomatic picture of apical periodontitis persists after well-performed endodontic therapy and the orthograde pathway is complex or blocked [16,17]. In addition, IR can be used in cases of incongruent endodontic therapy with excessive filling material beyond the apex and persistence of the apical lesion that cannot be resolved by a surgical approach [18], and when surgical retraction of the endodontic flap is contraindicated due to anatomic or accessibility limitations [19]. In addition, IR may be recommended for external root resorption when it is not possible to reach the apex [20], root perforations [21], root fractures, and complex roots [22]. In addition, IR may also be useful to treat teeth with developmental anomalies, such as fused teeth or teeth with a type C endodontic canal configuration [23].

IR has been proposed as an alternative procedure when endodontic and periapical surgical treatments have been unsuccessful or are contraindicated and when bone preservation is required for subsequent implant placement. Therefore, the present systematic review aimed primarily to estimate the survival rate of single-rooted and multi-rooted teeth after intentional replantation by assessing whether the number of roots could influence this and, secondarily, to compare the treatment results in upper and lower arch teeth.

## 2. Materials and Methods

The study protocol was developed, according to PRISMA (Preferred Reporting Items for Systematic Review and Meta-Analyses) guidelines [24,25], before the analysis.

The research question was formulated according to the PICO (Population, Intervention, Comparison, Outcome) strategy and the clinical question in the “PICO” format was “What is the survival rate of IR in single and multi-rooted permanent teeth?”, focusing on:

P (Population): Subjects undergone Intentional Replantation of permanent teeth
I (Intervention): Intentional Replantation of permanent teeth
C (Comparison): Single-rooted vs Multi-rooted replanted teeth
O (Outcome): Intentional Replantation survival rate

### 2.1. Search Strategy and Study Selection

A literature search was independently conducted by three reviewers (MP, AI, FDS), across PubMed/MEDLINE, Google Scholar, and the COCHRANE library databases and the gray literature, using the following keywords combined by Boolean operators: intentional replantation OR replanted teeth OR replanted tooth AND procedure OR technique.

Citations obtained through the literature search were recorded, duplicates were eliminated using EndNote, and titles and abstracts were independently screened by three reviewers (MP, FDS, GS). Available full-texts, compliant with inclusion and exclusion criteria, detailed below, were also independently reviewed for potentially eligible studies. Any disagreement between the reviewers was solved by discussion and consensus.

The inclusion criteria were:

- Source: studies published in the English language from January 1996 to 1 July 2022;
- Study design: case reports, case series, analytical observational studies, trials;
- Study population: subjects undergone IR (no age nor gender restrictions);
- Study intervention: IR of single-rooted and/or multi-rooted permanent teeth; and
- Study outcomes: IR reported clinical and/or patient-related outcomes.

The exclusion criteria were:

Source: studies published before 1996;

Study intervention: indication to treatment not specified; and

Study outcomes: IR clinical and/or patient-related outcomes not available.

No attempt to contact the authors was performed for missing information or full-text unavailability and, in case of disagreement, the evaluation of the majority was considered (two reviewers out of three).

Search and study selection was conducted for grey literature, as already described.

### 2.2. Data Extraction

A ten-question data extraction form was currently employed, by three independent reviewers (AI, GS, FDS), to record for each of the included study: source and design; participants’ age and gender; treated teeth; extra-alveolar time stay and environmental conditions management; IR indications, follow-up and reported outcomes, classified as IR success and failure, as reported by the authors in the included studies.

### 2.3. Data Synthesis and Statistical Analysis

Extracted data were synthesized according to the number of roots of replanted teeth, categorized as single- or multi-rooted.

Frequencies and percentages for categorical data were computed. A chi-square test with Yates correction was used to assess the association between teeth (single-rooted vs. multi-rooted) and dental arch (upper vs. lower). A standard statistical software package (SPSS, version 28.0; SPSS IBM, Armonk, New York, NY, USA) was used. The level of significance was set at *p* < 0.05.

### 2.4. Quality Assessment

Included studies were assessed for quality through the JBI (Joanna Briggs Institute) Critical Appraisal Tool, evaluating the risk of bias of the case reports and case series included [26].

## 3. Results

### 3.1. Study Selection

A total of 1556 records were retrieved from PubMed/MEDLINE (720 articles), Google Scholar (776 articles) and the COCHRANE library (60 articles). Of these, 904 were excluded because duplicates or the full text were not available. Of the remaining 652 articles, 130 were considered appropriate, but 70 were excluded because the full-text review did not reveal clinical cases treated with the technique IR. Finally, 60 articles were included in the qualitative analysis [27].

**Figure 1:**
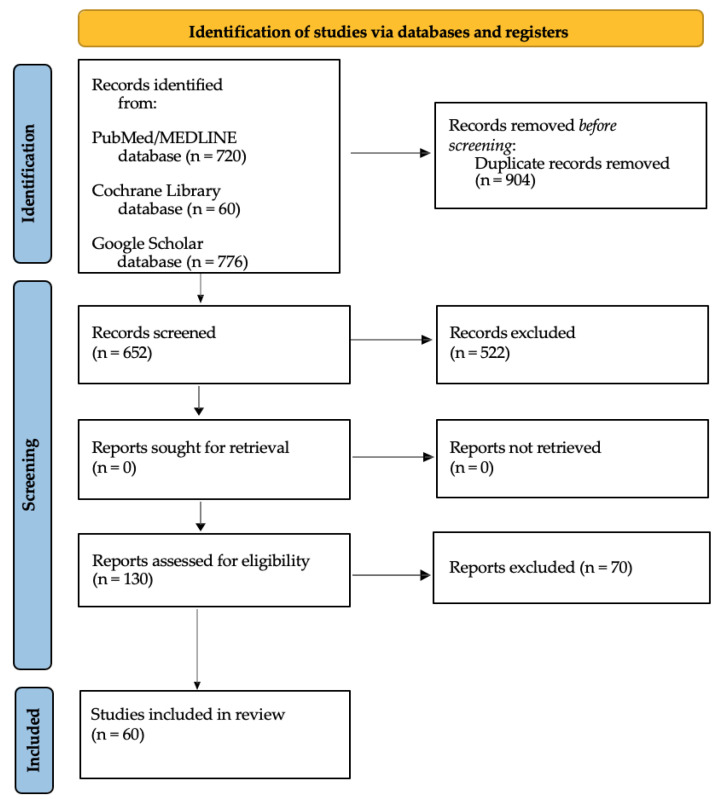
Study selection flowchart.

The flowchart for study selection is shown in Figure 1.

### 3.2. Studies Characteristics and Synthesis of the Reported Results

Sixty case reports and case series [28,29,30,31,32,33,34,35,36,37,38,39,40,41,42,43,44,45,46,47,48,49,50,51,52,53,54,55,56,57,58,59,60,61,62,63,64,65,66,67,68,69,70,71,72,73,74,75,76,77,78,79,80,81,82,83,84,85,86], compliant with the eligibility criteria, were included in the present systematic review, and detailed in Table 1; no observational studies or clinical trials were presently retrieved. The results of the risk of bias assessments of the included studies are reported in Table 2.

In total, 106 subjects, 48 males (45.2%) and 54 females (54.8%), between 7 and 86 years of age, with a mean age of 35.8, were treated with IR.

**Figure 2:**
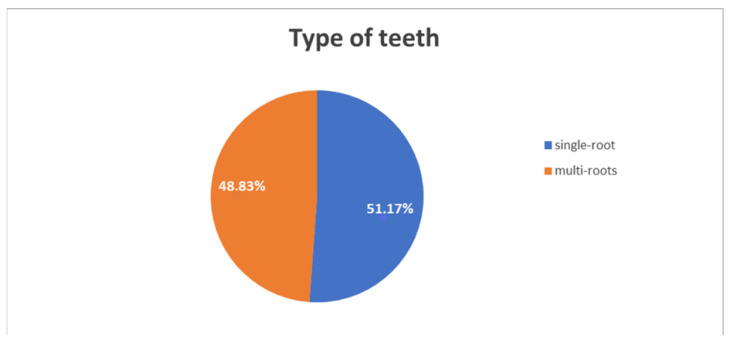
Type of teeth.

**Figure 3:**
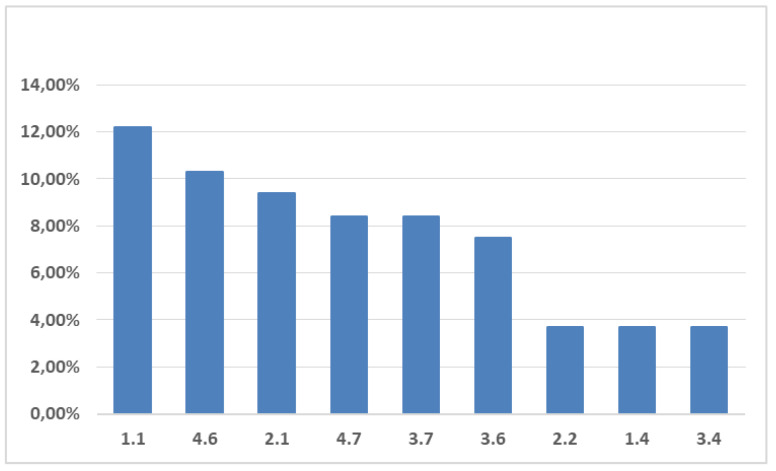
Percentages of treated teeth.

IR was performed on a total of 106 teeth, 56 (51.17%) single-rooted and 50 (48.9%) with multiple roots (Figure 2), with the upper right central incisors (12.2%) and the first mandibular right molars (10.3%) being the most treated, followed by central maxillary left incisor (9.4%), second mandibular right molars (8.4%), second mandibular left molars (8.4%), first mandibular left molars (7.5%), lateral maxillary left incisors (3.7%), first maxillary right premolars (3.7%) and first mandibular left premolars (3.7%) (Figure 3).

Extra-alveolar time stay was reported in 72 of 106 cases, corresponding to an average of 13.01 min. No data on the management of extra-alveolar environmental conditions and healing time could be retrieved.

The reported IR indications were: persistent periapical lesions in 77 (72.6%) cases; crown-root and root fractures in 19 (17.9%) replanted teeth; endodontic failure in 27 (25.5%) replanted teeth, six (22.2%) of them had perforation and 11 (40.7%) had intracanal instrument fracture; periodontitis in 13 (12.2%) cases; root resorption in five (4.7%) teeth and developmental anomaly with fused teeth in one case.

IR results were reported in all studies included in this systematic review. Treatment success was noted in 92 (86.7%) replanted teeth, with a mean follow-up of 26.8 months. Of the 56 (51.2%) single-rooted teeth replanted, six (10.7%) failed, with a survival rate of 89.3% at an average extraoral time of 12.48 min; of the 50 (48.9%) multi-rooted teeth replanted, eight (16%) failed, with a survival rate of 84% at an average extraoral time of 13.34 min (a minimum follow-up time of 3 months was considered). Of the 14 unsuccessful replantations, six had a single root (42% of failures) and eight were multi-rooted (58% of failures). Overall, seven (12.70%) replanted teeth from the upper arch (51.9%) and seven (13.7%) from the lower arch failed.

No statistically significant differences were found in the survival rates of replanted teeth between single- and multi-rooted teeth (*p* = 0.6) and between the upper and lower arches (*p* = 0.89).

## 4. Discussion

The present systematic review aimed, primarily, at the teeth survival rate following intentional replantation and, secondarily, to compare treatment outcomes in single- vs. multi-rooted teeth.

Despite the various IR indications reported in the literature, such as persistent apical periodontitis [16,17,18], incongruous endodontic therapy [17], inaccessible external root resorption [20], root perforations [21], complex root/coronal root fractures [21,22,23], and teeth with developmental anomalies, such as fused teeth, the procedure is considered a “last resort” to preserve natural teeth [1]. This consideration may be mainly due to the high risk of technical errors resulting from the numerous operative phases, which make the procedure highly operator-dependent and may explain the IR heterogeneous survival rates, which range from 80% to 100% in the literature [87]. However, a recent systematic review [88] described a survival rate of 88% IR, which is consistent with the current estimated survival rate of 86.7% IR. It is suggested that these results are closely related to the extra-alveolar time stay of the replanted teeth, which is considered to be a crucial factor as it is directly involved in the preservation of the PDL cells [18,19]. Indeed, the analysis of IR clinical cases included in the present systematic review revealed that the extra-alveolar time ranged from a minimum of 4 min [66,67,68,69,70,71,72,73,74,75,76,77,78,79,80] to a maximum of 30 min [35,36,37,38,39,40,41,42,43,44,45,46,47,48,49,50,51,52,53,54,55,56,57,58,59,60]. In particular, Jang et al. [89] reported higher survival rates for teeth replanted within 15 min compared with teeth replanted after an extra-alveolar time of more than 15 min. Nevertheless, high survival rates were also reported in cases with an extra-alveolar time > 15 min. Remarkably, however, the teeth in these cases were stored in a moist environment to preserve the viability of the PDL cells [3,7,8,11,12,13,18,19,90], suggesting that extra-alveolar stay time should be considered in the context of tooth conservation approaches. In this context, it has been previously suggested [8,10,91] that preservation of the tooth in an extra-oral humid environment, such as water, saline, and saliva, may positively influence the results of IR, making the procedure more predictable and thus supporting the hypothesis that the periodic submersion of the tooth in a bath of Hank’s balanced salt solution during the root resection phase may be the best approach to avoid root desiccation [91].

Moreover, high variability in root resection methods, filling materials, and socket manipulation were also noted. Although the length of root resection was rarely reported in the studies analyzed, most authors described a mean resection length of 1 to 3 mm [6]. Several restorative materials were listed in the reports, including mainly dental amalgam, followed by recently proposed intermediate restorative materials such as SuperEBA, MTA, and Endocem, and finally eugenol cement based on zinc oxide and glass ionomer [47]. Various approaches have also been found to manipulate the alveolus prior to tooth reinsertion. These include simple blood clot aspiration using suction instruments and/or irrigation with saline solution, as well as curettage of the alveolus with surgical instruments [22], which, however, may damage the fibers of the periodontal ligament still adhering to the alveolar walls, and affect, in turn, the success of IR, as mentioned above. Therefore, the recorded results show how the manipulation of the alveolus can have a crucial impact on the results of IR, which remains highly controversial [7,87]. According to Wu et al., if the reimplanted teeth are diagnosed with an acute or chronic apical abscess on preoperative examination, the risk of failure is 2.7 times higher than for teeth diagnosed with other conditions. This is because the presence of infection combined with chronic inflammation would lead to the destruction of the periodontal bone and PDL cells damage [92].

When the survival rates of IR were compared between single-rooted and multirooted teeth, no statistically significant differences were found. Therefore, it can be concluded that the number of roots of the replanted teeth has no significant influence on the results of the IR procedure. Nevertheless, special attention must be paid to the possible anatomical variations of the treated tooth root, especially pronounced curvatures. Therefore, Cone Beam Computed Tomography (CBCT) can be an essential tool in the diagnosis of anatomical variants, fractures, or discontinuities, which were previously based on a conventional, less sensitive 2D examination. However, because CBCT has only recently been introduced to support IR surgical planning, there are few case reports to date describing an IR planning phase using 3D reconstructions [93]. In addition, other recently introduced technologies, including ultrasonic devices and microscopy, may also both minimize the extra-alveolar time stay and improve treatment outcomes by reducing the duration of the IR procedure, invasiveness, and failure rates [94].

The main limitations of the study may be the exclusion of some databases (i.e., Scopus, LILACS, and EMBASE) from the electronic search and the inclusion of only case reports or case series, which are intrinsically characterized by low evidence and positive findings, that, along with the lack of data on the methods used to preserve the teeth in an extra-oral environment during the procedure, the heterogeneous approaches used to manipulate the alveolar socket, and the follow-up periods recorded, may make the interpretation of the results challenging. However, to the authors’ knowledge, this is the first study to investigate the possible role of the number of roots of the replanted teeth on treatment outcomes and to compare the survival rates of IR between single-rooted and multi-rooted teeth, even though the exact number of roots of multi-rooted teeth is not considered.

The results presented make it clear that IR can be considered a safe and predictable treatment for both single-rooted and multi-rooted teeth as long as all procedural phases are performed correctly [95]. Moreover, it seems evident that the success of IR also depends on the appropriateness of the treatment indications, suggesting the need for a comprehensive and multidisciplinary approach in complex cases [96,97] and supporting, once again that the choice of therapeutic strategy, even considering alternative procedures such as surgical extrusion and dental autotransplantation [98,99,100,101], should be based on the specific characteristics of each clinical case.

## 5. Conclusions

From the retrieved data, a survival rate of 86.7% was currently estimated for intentional replantation, and no statistically significant difference was found between single-rooted and multi-rooted replanted teeth, the survival rate of single-root implanted teeth was 89.3% while for multi-rooted reimplanted teeth it was 84%.

The reported results suggest that intentional replantation can be considered a safe therapeutic choice for both single-rooted and multi-rooted teeth, with a high survival rate and predictability, provided it is performed correctly and in accordance with basic biological principles, especially with regard to extra-oral environmental time.

## Figures and Tables

**Table 1:** Included studies characteristics: source, study participants’ gender and age; treated teeth, extra-alveolar time stay, and extra-alveolar environmental conditions management; IR indication(s), outcomes and follow-up. Abbreviations: y.o., years old; MR, multi-rooted; SR, single-rooted; IR, Intentional Replantation.

Source	Participants’ Gender Age	Treated Teeth	Extra-Alveolar Time Stay	Extra-Alveolar Conditions Management	IR Indication(s)	IR Outcomes	IR Follow-Up
Tang 1996 [28]	Male 29 y.o.	3.6 MR	-	-	Iatrogenic root furcation perforation	Survival	17 months
Poi 1999 [29]	Male 30 y.o.	4.5 SR	-	-	Instrument separation/Root perforation	Survival	8 years
Aqrabawi 1999 [30] Case 1	Female 46 y.o.	3.7 MR	15 min	-	Endodontic failure/Apical periodontitis	Survival	5 years
Aqrabawi Case 2	Female 38 y.o.	3.7 MR	20 min	-	Endodontic failure/Apical periodontitis	Survival	5 years
Benenati 2003 [31]	Female 45 y.o.	4.7 MR	-	-	Pain	Survival	16 years
Fariniuk 2003 [32]	Male 11 y.o.	1.1 SR	-	-	Crown-root fracture	Survival	3 years
Ward 2004 [33]	Female 68 y.o.	3.4 SR	15 min	-	Apical periodontitis	Survival	18 months
Shintani 2004 [34]	Male 7 y.o.	3.1 SR	10 min	-	Apical periodontitis with coronal fracture	Survival	5 years
Peer 2004 Case 1 [19]	Male 47 y.o.	3.5 SR	-	-	Apical periodontitis	Survival	30 months
Peer 2004 Case 3	Male 70 y.o.	3.2 SR	-	-	Apical periodontitis/sinus tract	Survival	4 years
Peer 2004 Case 4	Male 40 y.o.	3.7 MR	-	-	Apical periodontitis/sinus tract	Failure	7 years
BarattoFilho 2004 [35]	Male 36 y.o.	2.7 MR	15 min	-	Apical periodontitis/Instrument separation	Survival	5 years
Cotter 2006 [36]	Female 47 y.o.	3.1 SR	5 min	-	Apical periodontitis	Survival	1 years
Herrera 2006 [37]	Female 56 y.o.	4.6 MR	30 min	-	Apical periodontitis/endodontic failure	Survival	14 years
Martins [38]	Female 15 y.o.	2.1 SR	10 min	-	Traumatic avulsion	Survival	3 years
Penarrocha 2007 [39]	Female 20 y.o.	2.6 MR	5 min	-	Odontogenic maxillary sinusitis	Survival	2 years
Demir 2007 [40]	Male 45 y.o.	4.1 SR	-	-	Severe periodontitis	Survival	1 year
Sivolella 2008 [41]	Male 9 y.o.	1.2 SR	20 min	-	Double tooth	Survival	6 years
Wang 2008 [42]	Female 8 y.o.	1.1 SR	15 min	-	Complicated crown-root fracture	Failure	3 months
Al-Hezaimi 2009 [43]	Female 15 y.o.	1.2 SR	-	-	Pulp necrosis with suppurative apical periodontitis	Survival	4 years
Bittencourt 2009 [44]	Male 9 y.o.	2.1 SR	-	-	Complicated crown-root fracture	Survival	2 years
Ozer 2010 [45] Case 1	Male 36 y.o.	1.1 SR 1.2 SR	(1.1) 12 min (1.2) 16 min	-	Vertical root fracture	Survival	2 years
Ozer Case 2	Female 25 y.o.	2.2 SR	18 min	-	Vertical root fracture	Survival	2 years
Ozer Case 3	Male 32 y.o.	1.3 SR	24 min	-	Vertical root fracture	Survival	2 years
Hsiang Lu 2011 [46]	Male 50 y.o.	4.6 MR	13 min	-	Apical periodontitis	Survival	3 months
Unver 2011 [47]	Female 41 y.o.	1.4 MR	25 min	-	Vertical fracture	Survival	36 months
Kim 2011 [48] Case 1	Female 23 y.o.	1.1 SR 2.1 SR 2.2 SR	-	-	Complicated crown-root fractures	Failure Survival Survival	90 months
Kim 2011 Case 2	Female 27 y.o.	2.1 SR	-	-	Complicated crown-root fracture	Survival	24 months
Moura 2012 [49]	Female 11 y.o.	1.1 SR	-	-	Complicated crown-root fracture	Failure	2 years
Dogan 2013 [50]	Female 9 y.o	2.1 SR	28 min	-	complicated crown-root fracture	Survival	3 years
Shin 2013 [51]	Male 39 y.o.	4.6 MR	17 min	-	Apical periodontitis	Survival	9 months
Yuan 2013 [52]	Female 11 y.o.	2.1 SR	-	-	Complicated crown-root fracture	Survival	3.5 years+
Nagappa 2013 [53] Case 1	Female 18 y.o.	1.1 SR	-	-	Severe periodontitis	Failure	3 months
Nagappa Case2	Male 24 y.o.	2.1 SR	-	-	Severe periodontitis	Survival	14 months
Kumar 2013 [54]	Male 26 y.o.	2.2 SR	8 min	-	Apical periodontitis/endodontic failure	Survival	1 year
MoradiMajd 2012 [55]	Female 44 y.o.	3.5 SR	-	-	Apical periodontitis/necrotic tooth	Survival	1 year
Subay 2014 [56]	Female 45 y.o.	4.3 SR	14 min	-	Apical periodontitis/Instrument separation	Survival	24 months
Asgary 2014 [57] Case 1	Male 25 y.o.	4.6 MR	14 min	-	Apical periodontitis	Survival	23 months
Asgary Case 2	Male 45 y.o.	3.4 SR	10 min	-	Apical periodontitis	Survival	30 months
Asgary Case 3	Male 41 y.o.	4.7 MR	8 min	-	Apical periodontitis	Survival	24 months
Asgary Case 4	Male 23 y.o.	4.6 MR	12 min	-	Apical periodontitis	Survival	15 months
Asgary Case 5	Female 46 y.o.	4.7 MR	8 min	-	Apical periodontitis	Survival	27 months
Asgary Case 6	Female 31 y.o.	4.7 MR	9 min	-	Apical periodontitis	Survival	12 months
Asgary Case 7	Female 30 y.o.	1.4 MR	10 min	-	Apical periodontitis	Failure	18 months
Asgary Case 8	Female 36 y.o.	3.6 MR	13 min	-	Apical periodontitis	Survival	14 months
Asgary Case 9	Male 48 y.o.	4.7 MR	14 min	-	Apical periodontitis	Survival	16 months
Asgary Case 10	Female 24 y.o.	4.6 MR	14 min	-	Apical periodontitis	Survival	8 months
Asgary Case 11	Female 43 y.o.	2.6 MR	14 min	-	Apical periodontitis	Survival	17 months
Asgary Case 12	Male 34 y.o.	3.4 SR	12 min	-	Apical periodontitis	Survival	15 months
Asgary Case 13	Female 29 y.o.	3.6 MR	10 min	-	Apical periodontitis	Survival	11 months
Asgary Case 14	Male 63 y.o.	3.6 MR	14 min	-	Apical periodontitis	Survival	12 months
Asgary Case 15	Male 31 y.o.	1.7 MR	13 min	-	Apical periodontitis	Survival	10 months
Asgary Case 16	Female 46 y.o.	4.6 MR	14 min	-	Apical periodontitis	Survival	8 months
Asgary Case 17	Female 40 y.o.	4.6 MR	12 min	-	Apical periodontitis	Failure	8 months
Asgary Case 18	Female 27 y.o.	4.7 MR	13 min	-	Apical periodontitis	Survival	20 months
Asgary Case 19	Female 41 y.o.	3.6 MR	10 min	-	Apical periodontitis	Survival	12 months
Asgary Case 20	Male 37 y.o.	4.7 MR	10 min	-	Apical periodontitis	Survival	9 months
Asgari 2014 [58]	Female 28 y.o.	1.4 MR 1.5 SR	8 min	-	Apical periodontitis	Survival	2 years
MoradiMajd 2014 [59]	Female 44 y.o.	4.5 SR	-	-	Iatrogenic perforation	Failure	1 year
Penarrocha Diego 2014 [60]	Male 51 y.o.	1.7 MR	30 min	-	Follicular cyst	Survival	12 months
Tsesis 2014 [61]	Female 20 y.o.	4.7 MR	8 min	-	Paraesthesia	Survival	4 years
Keceli 2014 [62]	Female 20 y.o.	3.2 SR	6 min	-	Severe periodontitis	Survival	15 months
Pruthi 2015 [63]	Male 28 y.o.	1.1 SR	15 min	-	External root resorption	Survival	18 months
DeeptiDua 2015 [64]	Male 23 y.o.	1.1 SR	20 min	-	Complicated crown-root fracture	Survival	3 years
Forero-Lopez 2015 [65]	Male 25 y.o.	1.2 SR	8 min	-	Apical periodontitis	Survival	3 months
Garrido 2016 [66]	Female 50 y.o.	1.1 SR	4 min	-	Endo-periodontal disease	Survival	1 year
Abu-Hussein Muhamad 2016 [67]	Female 45 y.o.	1.7 SR	20 min	-	Apical periodontitis/Instrument separation	Survival	15 years
Oishi 2017 [68]	Male 7 y.o.	1.1 SR	-	-	Transverse root fracture/Endo-periodontal disease	Survival	5 years
Grzanich 2017 [69] Case 1	Female 64 y.o.	3.1 SR	-	-	Apical periodontitis/Instrument separation	Survival	28 months
Grzanich Case 2	Male 35 y.o.	1.4 MR	-	-	Apical periodontitis/endodontic	Survival	2 years
Grzanich Case 3	Female 86 y.o.	1.8 SR	-	-	Apical periodontitis/vertical root fracture	Survival	2 years
Faghihian 2017 [70]	Male 10 y.o.	1.1 SR	4 min	-	complicated crown-root fracture	Survival	18 months
Maniglia-Ferreira 2017 [71]	Male 7 y.o.	1.1 SR	15 min	-	Traumatic avulsion	Survival	3 years
Thaore 2017 [72]	Male 23 y.o.	3.7 MR	10 min	-	Apical periodontitis/Instrument separation	Survival	1 year
Asgari 2018 [73]	Female 22 y.o.	4.6 MR	7 min	-	Apical periodontitis	Survival	2 months
Zafar 2018 [74]	Female 30 y.o.	2.6 MR	15 min	-	Apical periodontitis/Instrument separation	questionable	4 weeks
Saeed Kazi 2018 [75]	Male 35 y.o.	4.7 MR	10 min	-	Root perforation	Survival	4 months
Krug 2019 [76]	Male 37 y.o.	1.1 SR	12 min	-	External cervical resorption	Survival	2.5 years
Deshpande 2019 [77]	Female 23 y.o.	1.6 MR	10 min	-	Apical periodontitis/Instrument separation	Survival	2 years
Teng Kai Ong 2019 [78]	Male 27 y.o.	1.7 MR	15 min	-	Symptomatic periradicular periodontitis	Failure	10 months
Hao Yan 2019 [79] case1	Male 37 y.o.	2.2 SR	7 min	-	Apical periodontitis	Survival	18 months
Hao Yan 2019 case2	Male 30 y.o.	1.2 SR	6 min	-	Apical periodontitis	Survival	15 months
Hao Yan 2019 case3	Female 27 y.o.	1.2 SR	6 min	-	Apical periodontitis	Survival	12 months
Cunliffe 2020 [80] Case 1	Male 33 y.o.	4.1 SR	15 min	-	Instrument separation/Root perforation	Survival	6 months
Cunliffe Case 2	Female 45 y.o.	3.4 SR	15 min	-	Apical periodontitis with missed anatomy	Failure	3 months
Cunliffe Case 3	Female 52 y.o.	4.6 MR	15 min	-	Apical periodontitis with over-filled	Failure	3 months
Cunliffe Case 4	Female 57 y.o.	4.4 SR	4 min	-	Apical periodontitis/pain	Survival	1 year
Cunliffe Case 5	Female 42 y.o.	3.6 MR	-	-	Apical periodontitis	Survival	3 months
Cunliffe Case 6	Male 64 y.o.	2.1 SR	15 min	-	External root resorption	Survival	4 months
Cunliffe Case 7	Female 76 y.o.	3.7 MR	-	-	Apical periodontitis with sclerosed canals	Failure	1 year
Cunliffe Case 8	Male 53 y.o.	3.7 MR	-	-	Pulpal floor perforation	Survival	3 months
Cunliffe Case 9	Male 50 y.o.	2.1 SR	-	-	Internal root resorption	Survival	15 months
Cunliffe Case 10	Female 64 y.o.	3.7 MR	15 min	-	Instrument separation	Survival	6 months
Cunliffe Case 11	Female 45 y.o.	3.7 MR	-	-	Apical periodontitis with over-filled	Survival	28 months
Cunliffe Case 12	Male 45 y.o.	4.5 SR	-	-	Apical periodontitis	Survival	9 months
Cunliffe Case 13	Female 39 y.o.	3.6 MR	-	-	Apical periodontitis with procedural errors	Failure	3 months
Asgary 2019 [81]	Female 28 y.o.	3.7 MR	10 min	-	Apical periodontitis/endodontic failure	Survival	1 year
Fujii 2020 [82]	Female 30 y.o.	1.6 MR	15 min	-	Instrument separation	Survival	1 year
Ganapathy 2020 [83]	Male 10 y.o.	2.1 SR	-	-	Complicated crown-root fracture	Survival	2 years
Lodha 2020 [84]	Female 28 y.o.	4.6 MR	10 min	-	Apical periodontitis/Instrument separation	Survival	8 months
Yang 2021 [85]	Male 20 y.o.	1.5 SR	15 min	-	Apical periodontitis with internal root resorption and root fracture	Survival	2 years
Shekhawat 2021 [86]	Male 13 y.o.	3.6 MR	15 min	-	Apical periodontitis	Survival	12 months

**Table 2:** JBI Critical Appraisal Tool. Abbreviations: JBI Joanna Briggs Institute; “Q1–Q11 indicate questions 1 to 11 based on the JBI risk assessment”. **Questions:** “1. Is the review question clearly and explicitly stated? 2. Were the inclusion criteria appropriate for the review question? 3. Was the search strategy appropriate? 4. Were the sources and resources used to search for studies adequate? 5. Were the criteria for appraising studies appropriate? 6. Was critical appraisal conducted by two or more reviewers independently? 7. Were there methods to minimize errors in data extraction? 8. Were the methods used to combine studies appropriate? 9. Was the likelihood of publication bias assessed? 10. Were recommendations for policy and/or practice supported by the reported data? 11. Were the specific directives for new research appropriate?”. x: no; √: yes; ?: questionable.

Source	Q1	Q2	Q3	Q4	Q5	Q6	Q7	Q8	Q9	Q10	Q11	%Yes	Risk
Tang 1996	x	√	√	√	x	√	√	√	?	x	x	55%	moderate
Poi 1999	x	?	x	x	x	√	√	√	x	x	x	27%	high
Aqrabawi 1999	√	√	?	x	x	√	√	√	x	x	?	45%	high
Benenati 2003	x	√	√	x	√	√	√	√	?	x	x	55%	moderate
Fariniuk 2003	√	√	√	x	x	√	√	√	?	x	x	55%	moderate
Ward 2004	x	√	√	x	x	√	√	√	?	x	√	55%	moderate
Shintani 2004	x	√	√	√	x	√	√	√	x	x	?	55%	moderate
Peer 2004	√	√	√	?	x	√	√	√	x	x	x	55%	moderate
BarattoFilho 2004	√	?	√	√	√	√	√	√	√	x	√	81%	low
Cotter 2006	x	x	√	√	x	√	√	√	√	x	?	55%	moderate
Herrera 2006	√	√	?	x	x	√	√	√	?	x	x	45%	high
Martins	√	√	x	?	x	√	√	√	x	x	?	45%	high
Penarrocha 2007	√	√	√	√	?	√	√	√	√	x	√	81%	low
Demir 2007	√	?	√	√	√	√	√	√	√	x	√	81%	low
Sivolella 2008	x	?	x	x	x	√	√	√	x	x	x	27%	high
Wang 2008	x	x	√	√	x	√	√	√	?	x	√	55%	moderate
Al-Hezaimi 2009	x	?	√	√	x	√	√	√	√	x	x	55%	moderate
Bittencourt 2009	x	x	√	√	x	√	√	√	√	x	?	55%	moderate
Ozer 2010	x	x	√	√	x	√	√	√	?	x	√	55%	moderate
Hsiang Lu 2011	x	?	√	√	x	√	√	√	√	x	x	55%	moderate
Unver 2011	√	√	?	√	√	√	√	√	√	x	√	81%	low
Kim 2011	√	√	x	?	x	√	√	√	x	x	?	45%	high
Moura 2012	√	√	?	x	x	√	√	√	x	x	?	45%	high
Dogan 2013	x	x	?	x	x	√	√	√	x	x	x	27%	high
Shin 2013	x	x	√	√	x	√	√	√	?	x	√	55%	moderate
Yuan 2013	x	x	√	√	x	√	√	√	?	x	√	55%	moderate
Nagappa 2013	x	x	√	√	x	√	√	√	?	x	√	55%	moderate
Kumar 2013	√	√	√	√	?	√	√	√	√	x	√	81%	low
Moradi Majd 2014	x	x	√	x	√	√	√	√	?	x	√	55%	moderate
Subay 2014	x	√	x	√	x	√	√	√	√	x	?	55%	moderate
Asgary 2014	x	x	√	√	x	√	√	√	?	x	√	55%	moderate
Asgari 2014	x	?	x	x	x	√	√	√	x	x	x	27%	high
Moradi Majd 2014	x	x	√	x	√	√	√	√	?	x	√	55%	moderate
Penarrocha Diego 2014	x	√	x	√	x	√	√	√	√	x	?	55%	moderate
Tsesis 2014	x	x	√	√	x	√	√	√	?	x	√	55%	moderate
Keceli 2014	x	x	?	x	x	√	√	√	x	x	x	27%	high
Pruthi 2015	x	√	x	√	x	√	√	√	√	x	?	55%	moderate
Deepti Dua 2015	x	x	√	√	x	√	√	√	?	x	√	55%	moderate
Forero-Lopez 2015	x	?	x	x	x	√	√	√	x	x	x	27%	high
Garrido 2016	x	x	√	x	√	√	√	√	?	x	√	55%	moderate
Abu-Hussein Muhamad 2016	x	√	x	√	x	√	√	√	√	x	?	55%	moderate
Oishi 2017	x	x	√	x	√	√	√	√	?	x	√	55%	moderate
Grzanich 2017	x	?	x	x	x	√	√	√	x	x	x	27%	high
Faghihian 2017	√	√	√	√	?	√	√	√	√	x	√	81%	low
Maniglia-Ferreira 2017	√	√	?	X	x	√	√	√	x	x	?	45%	high
Thaore 2017	x	x	√	x	√	√	√	√	?	x	√	55%	moderate
Asgari 2018	x	√	x	√	x	√	√	√	√	x	?	55%	moderate
Zafar 2018	√	√	x	?	x	√	√	√	x	x	?	45%	high
Saeed Kazi 2018	x	x	√	x	√	√	√	√	?	x	√	55%	moderate
Krug 2019	√	√	√	√	x	√	√	√	√	?	√	81%	low
Deshpande 2019	√	√	√	?	√	√	√	√	√	x	√	81%	low
Teng Kai Ong 2019	√	√	x	?	x	√	√	√	x	x	?	45%	high
Hao Yan 2019	√	√	?	x	x	√	√	√	x	x	?	45%	high
Cunliffe 2020	√	√	√	√	?	√	√	√	√	x	√	81%	low
Asgary 2019	√	x	√	?	x	√	√	√	x	x	?	45%	high
Fujii 2020	x	√	x	√	x	√	√	√	√	x	?	55%	moderate
Ganapathy 2020	x	x	x	x	?	√	√	√	x	x	x	27%	High
Lodha 2020	x	x	?	x	x	√	√	√	x	x	x	27%	high
Yang 2021	x	√	x	√	x	√	√	√	√	x	?	55%	moderate
Shekhawat 2021	√	√	√	√	?	√	√	√	√	x	√	81%	low

## Data Availability

Medline/PubMed, Cochrane databases and Google Scholar.
